# Dissociating retrieval success from incidental encoding activity during emotional memory retrieval, in the medial temporal lobe

**DOI:** 10.3389/fnbeh.2014.00177

**Published:** 2014-06-03

**Authors:** Andrea T. Shafer, Florin Dolcos

**Affiliations:** ^1^Centre for Neuroscience, University of AlbertaEdmonton, AB, Canada; ^2^Social, Cognitive, Personality, and Emotional Neuroscience Laboratory, Psychology Department, Neuroscience Program, Beckman Institute for Advanced Science and Technology, University of Illinois at Urbana-ChampaignIL, USA

**Keywords:** affect, recollection, episodic memory, successful retrieval, incidental learning

## Abstract

The memory-enhancing effect of emotion has been linked to the engagement of emotion- and memory-related medial temporal lobe (MTL) regions (amygdala-AMY; hippocampus-HC; parahippocampus-PHC), during both encoding and retrieval. However, recognition tasks used to investigate the neural correlates of retrieval make it difficult to distinguish MTL engagement linked to retrieval success (RS) from that linked to incidental encoding success (ES) during retrieval. This issue has been investigated for retrieval of non-emotional memories, but not for emotional memory retrieval. To address this, we used event-related functional MRI in conjunction with an emotional distraction and two episodic memory tasks (one testing memory for distracter items and the other testing memory for new/lure items presented in the first memory task). This paradigm allowed for dissociation of MTL activity specifically linked to RS from that linked to both RS and incidental ES during retrieval. There were two novel findings regarding the neural correlates of emotional memory retrieval. First, greater emotional RS was identified bilaterally in AMY, HC, and PHC. However, AMY activity was most impacted when accounting for ES activity, as only RS activity in left AMY was dissociated from ES activity during retrieval, whereas portions of HC and PHC showing greater emotional RS were largely uninvolved in ES. Second, an earlier and more anteriorly spread response (left AMY and bilateral HC, PHC) was linked to greater emotional RS activity, whereas a later and more posteriorly localized response (right posterior PHC) was linked to greater neutral RS activity. These findings shed light on MTL mechanisms subserving the memory-enhancing effect of emotion at retrieval.

## Introduction

Investigations on the impact of emotion on memory have shown that emotion enhances memory (Bradley et al., 1992; Christianson, 1992; Chiu et al., 2013), and that this enhancement is associated with increased engagement of emotion (amygdala, AMY) and memory (hippocampus, HC and parahippocampus, PHC) related medial temporal lobe (MTL) regions. This increased engagement is observed during both encoding (Dolcos et al., 2004; McGaugh, 2004; Dolcos and Denkova, 2008; Murty et al., 2011) and retrieval (Sharot et al., 2004; Dolcos et al., 2005; Kensinger and Schacter, 2005; Sergerie et al., 2006; Smith et al., 2006); reviewed in Dolcos et al. (2012). While the MTL's role in encoding success (ES) operations contributing to the memory-enhancing effect of emotion has been well documented, open questions still remain concerning its role in emotional memory retrieval. One unclear aspect concerns the dissociation between neural activity linked to retrieval success (RS) processes and activity associated with encoding processes that occur during retrieval. Due to the nature of recognition memory tasks used to study the neural correlates of memory retrieval, it is unclear whether MTL regions identified as being associated with retrieval processes are unique to retrieval or are common to both successful retrieval and incidental encoding that occurs during retrieval. The present study addressed this issue by using functional magnetic resonance imaging (fMRI) in conjunction with an experimental design that allowed for the dissociation of MTL involvement in retrieval success from incidental encoding success, during the retrieval of emotional memories.

Investigations of MTL activity associated with incidental memory formation during non-emotional memory retrieval (Stark and Okado, 2003) found that MTL regions associated with neutral retrieval success largely overlapped with those involved in the incidental encoding of lure items. Even though a large amount of overlap was found, specificity within the HC was also found such that areas in the HC were identified as being associated with retrieval success after accounting for incidental encoding during retrieval. However, to our knowledge similar investigations have not been performed during emotional memory retrieval. Therefore, it remains unclear whether or not activity linked to the memory-enhancing effect of emotion identified in MTL-based emotion and memory regions during retrieval can be distinguished from activity related to the memory-enhancing effect of emotion associated with incidental memory formation during retrieval. Hence, the first goal of the present investigation was to address this issue by identifying MTL activity specifically related to the successful retrieval of emotional memories that does not contribute to incidental encoding during emotional memory retrieval.

Recognition memory tasks involve various aspects of processing, including retrieval operations *per se*, re-encoding/consolidation of retrieved memories, and incidental encoding of new information presented as lures. The focus of the present investigation is to distinguish between MTL areas subserving memory operations that contribute to the successful retrieval of information from MTL areas involved in incidental memory formation during retrieval. It should be noted that in the context of the present investigation we refer to “encoding operations” from a mnemonic not perceptual perspective. The former refer to memory-specific processing that leads to the formation of new memories, whereas the latter refer to general perceptual processing that occurs regardless of subsequent memory effects. There are currently two methodological approaches regarding the identification of the neural correlates of retrieval success. One compares activity for old items correctly identified as old (Hits) and activity for old items incorrectly identified as new (Misses), and the other compares activity for Hits and new items correctly identified as new (Correct Rejections, CR).

We defined retrieval success activity as resulting from the comparison between Hits and Misses, because the comparison between Hits and CR makes it difficult to distinguish between various aspects of processing during retrieval. For example, if a brain region is involved in both retrieval success *per se* and incidental encoding success during retrieval, then the incidental encoding success activity in response to a lure item that was correctly rejected may equate the retrieval success activity in response to an Old item that was remembered. In this situation a brain region would erroneously show no involvement in retrieval success activity, because it contributes to both aspects of processing. This has previously been shown for the involvement of MTL regions in non-emotional memory retrieval and incidental encoding (Stark and Okado, 2003).

One way to identify incidental encoding success activity, and dissociate it from retrieval success activity, is to use a second subsequent memory task (second retrieval task) and compare brain activity for Misses that are subsequently remembered to Misses that remain forgotten. However, this contrast cannot control for the effects associated with repeated presentation of these items, which may eventually lead to their encoding into memory. Therefore, it is difficult to determine if later memory for Misses that were initially forgotten during the first retrieval task and then remembered during the second retrieval task is due to successful encoding during the first retrieval or due to a repeated exposure effect where the signal for a particular item may finally surpass the criteria necessary for an old response.

To avoid repeated presentation, we favor an alternative way of identifying and dissociating incidental encoding success activity from retrieval success activity. This involves comparison of activity for new items/lures presented during the first retrieval task that are then remembered or forgotten in a subsequent memory task (second retrieval task). In this regard, incidental encoding success activity during retrieval refers to the successful encoding of items presented as lures during the first retrieval task. Using this approach allows for the separation of retrieval success activity, obtained by comparing Hits and Misses, from incidental encoding success activity resulted from the contrast between the remembered lures and forgotten lures (in the second retrieval task).

Previous investigations of the neural mechanisms of emotional memory have pointed to spatial and temporal dissociations. Regarding the former, fMRI investigations have identified an anterior-posterior dissociation in the MTL, with emotional memory involving more anterior regions (AMY and anterior HC, PHC regions) and neutral memory involving more posterior regions (middle to posterior HC and PHC) (Dolcos et al., 2004; Sharot et al., 2004; Kensinger and Schacter, 2005; Dougal et al., 2007). Regarding temporal dissociations, event-related potential (ERP) studies have pointed to earlier memory-related processing contributing to the memory-enhancing effect of emotion compared to that contributing to non-emotional memory (Dolcos and Cabeza, 2002). Although this finding is consistent with previous research highlighting faster processing of emotional information (Dolcos and Cabeza, 2002; Larson et al., 2006; Mendez-Bertolo et al., 2013), it is uncertain if these timing differences can also be observed in the BOLD response within the MTL during retrieval, especially given that the temporal resolution offered by fMRI is less than ideal for determining the temporal characteristics of cognitive processes. Nevertheless, when using fMRI, some information concerning their timing may still be gleaned (Siegle et al., 2002; Larson et al., 2006; Daselaar et al., 2008; Schuyler et al., 2012). For example, emotional and neutral retrieval activity may possess similar magnitudes, but time to peak onset of those magnitudes may differ, thus revealing dissimilarities between the associated neural mechanisms of these processes that would otherwise remain hidden when not considering the time course of the BOLD response. To that end, the second goal of the present investigation was to explore the possibility of differences in the time course of the BOLD response for emotional and neutral retrieval activity in the MTL.

**Figure 1:**
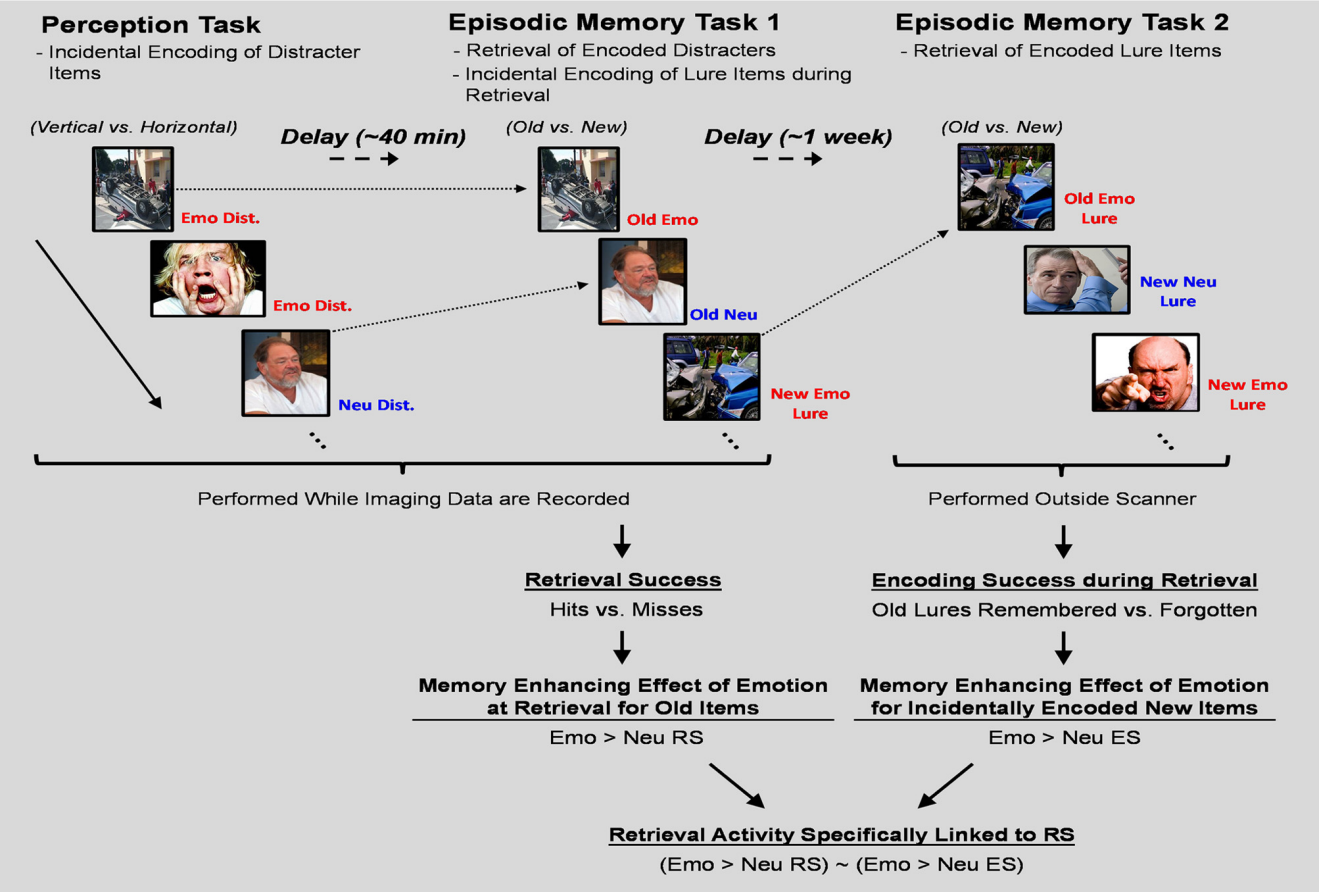
**Diagram of the experimental paradigm**. A novel experimental paradigm was implemented to investigate the relationship between retrieval success activity and incidental encoding success activity during retrieval. The perception task required participants to determine the orientation of vertically or horizontally presented pictures with emotional, neutral, or no distraction. Immediately following completion of the perception task, memory for a subset of the pictures presented as distracters during the task was tested in a surprise episodic memory task (EM-1). EM-1 allowed for the examination of the neural correlates of retrieval success of the distracter items incidentally encoded during the perception task, and also served as the “study” phase for emotional and neutral lure items. Approximately 1 week following the completion of the perception and EM-1 tasks, participants completed another surprise episodic memory task (EM-2), which tested memory for the emotional and neutral lure items used during EM-1. In both memory tasks participants were instructed to determine if the pictures were from the previous task (Old) or never seen before (New). Old/New decisions were followed by confidence ratings (1 = low, 2 = medium, 3 = high). Separation of RS from incidental ES activity was obtained by defining RS as the contrast between Hits and Misses and incidental ES as the contrast between Remembered and Forgotten Lure items. Emo, Emotional; Neu, Neutral; Dist., Distracter; RS, Retrieval Success; ES, Encoding Success

These issues were addressed by using an experimental design in which participants sequentially performed three tasks to identify and compare the neural correlates of retrieval success to those associated with incidental encoding success during retrieval. First, participants performed a perception task that served as the “study phase” for the items used to examine the neural correlates of retrieval. Then, immediately following the perception task, participants performed an episodic memory task that served as the “test phase” for the items used to examine the neural correlates of retrieval as well as the “study phase” for lure items used to identify the neural correlates of incidental encoding during retrieval. Lastly, participants performed another episodic memory task that served as the “test phase” for the lures that were presented during the first episodic memory task (see Figure 1). For the current investigation we restricted our analyses to regions within the MTL. This was done for three reasons. First, MTL engagement in memory processes is among the most systematic findings in the literature regarding the neuroscience of memory; hence, we targeted this region due to its reliable involvement in the processes under investigation. Second, while other non-MTL brain regions (e.g., frontal and parietal cortices) are also contributing to the memory-enhancing effect of emotion, their involvement tends to be mediated by the contribution of other processes, such as attention, working memory, and semantic memory (Dolcos et al., 2012; Shafer and Dolcos, 2012). In contrast, MTL regions are less susceptible to such influences due to their relatively automatic engagement during the encoding and retrieval of emotional memories (Ritchey et al., 2011; Shafer and Dolcos, 2012). Third, we primarily sought to build on the existing literature concerning the present research topic, where there was focus only on MTL-based memory regions for non-emotional memories (Stark and Okado, 2003).

Based on the extant evidence we made the following three predictions: Concerning the first main goal and consistent with previous research examining the influence of emotion on memory, we predicted enhanced memory for emotional relative to neutral items, during both memory tasks. Regarding the fMRI findings and based on earlier research for non-emotional memory (Stark and Okado, 2003), we predicted that the memory-enhancing effect of emotion at retrieval would be at least partially accounted for by activity related to memory-enhancing effect of emotion associated with incidental encoding during retrieval. Concerning the second main goal, and based on previous findings showing that encoding processes associated with the memory-enhancing effect of emotion occur earlier (Dolcos and Cabeza, 2002) and influence more anterior MTL regions (Dolcos et al., 2004), we explored the possibility that similar differences could also be identified at retrieval, with emotional retrieval success activity occurring earlier and in more anterior MTL regions than the neutral retrieval success activity.

## Methods

### Participants

Data from a group of 17 healthy young adults [19–33 years of age (*M* = 23.11, *SD* = 4.01); 10 females; all right-handed] were analyzed for the present investigation. Data from all 17 participants was used to examine the influence of emotion on immediate memory. Data from 10 participants (19–33 years of age, *M* = 24.6, *SD* = 4.53, 7 female) was used to examine the influence of emotion on delayed memory in order to examine incidental encoding during retrieval. Participants were recruited from the Edmonton City area, provided written informed consent before participating, and were reimbursed for their participation. The experimental protocol was approved for ethical treatment of the human participants by the Institutional Health Research Board.

### Tasks and stimuli

Each participant performed a perceptual orientation discrimination task and an episodic memory task (EM-1), while brain imaging data were collected using fMRI (Shafer and Dolcos, 2012; Shafer et al., 2012). One-week following the completion of these two tasks, participants also performed a delayed episodic memory task (EM-2) for items that were presented as lures during the immediate episodic memory task (see task diagram illustrated in Figure 1). In the perceptual orientation discrimination task, participants made decisions on the orientation of vertical and horizontal pictures with negative and neutral content. In the memory task immediately following the perception task, participants made decisions about whether emotional and neutral pictures had been presented during the perception task (Old) or they had not been seen before (New). In the delayed memory task, performed 5–7 days after the completion of the first two tasks, participants made decisions about whether or not emotional and neutral pictures were presented as lures during the immediate episodic memory task or were New items that had not been seen before. All of the emotional and neutral pictures were selected from the International Affective Pictures System (Lang et al., 2008), based on normative arousal and valence ratings and from in-house pictures used in previous studies (Yamasaki et al., 2002; Dolcos and McCarthy, 2006). For each individual task, valence and arousal scores were significantly different for emotional and neutral pictures. Notably, the arousal and valence scores did not differ across tasks within emotional or neutral picture categories. Since our main goal focused on effects associated with the overall emotional charge, rather than on identifying the relative contribution of basic emotional dimensions (arousal vs. valence), the present results cannot distinguish between the contribution of these two affective dimensions to the observed effects.

#### Perception task

The stimuli and design of the perception task are identical to those described previously (Shafer et al., 2012). Briefly, the perception task manipulated the attentional demand necessary to determine the orientation of vertically or horizontally presented pictures that contained emotional (negative), non-emotional (neutral), or no distraction (scrambled). The mean arousal (1 = Lowest/9 = Highest) and valence (1 = Very Negative, 5 = Neutral, 9 = Very Positive) scores for the 224 pictures used during the perception task, respectively, were as follows: 5.9/2.75, for emotional pictures; 3.35/5.05 for neutral pictures. Participants were instructed to determine the orientation of the rectangles, to maintain focus on the orientation task, and to respond as accurately and quickly as possible.

#### Episodic memory task 1 (EM-1)

The stimuli and design of the immediate episodic memory task are identical to those described previously (Shafer and Dolcos, 2012). Briefly, EM-1 was a surprise memory task for items that were presented as distracters during the perception task. This task consisted of 160 pictures, which were a sub-set of the 224 pictures presented in the initial perception task. These Old images were pseudo-randomized with 80 New pictures (40 emotional, 40 neutral) that were selected on normative arousal and valence scores and semantic content from the same picture databases used for the perception task. The average normative arousal and valence scores for Old and New emotional and neutral pictures for the first episodic memory task were as follows: 5.93/2.63 for emotional Old pictures; 5.95/2.66 for emotional New pictures; 3.41/5.04, for neutral Old pictures; and 3.41/5.02 for neutral New pictures. To ensure a minimum retention interval for the memory-enhancing effect of emotion to occur, a minimum delay of 20 min between the initial encoding and first retrieval task was imposed (Kleinsmith and Kaplan, 1963). Participants were randomly assigned one of two run orders which allowed for a similar delay period between encoding and retrieval. This was essential to the overall design of the paradigm as Old stimuli in the first episodic memory task were pseudo-randomized based on when they appeared in the perception task. This resulted in a delay of approximately 40 min between the encoding and retrieval of a stimulus. For example, if a picture was presented in the first run of the perception task, then it would be presented in either the first or second run of the recognition task. Likewise, if a stimulus was presented in the last run of the perception task then it was presented in the second to last or last run of the recognition task.

#### Episodic memory task 2 (EM-2)

Approximately 1-week (Range = 5–7 days; Mean = 6.8) following the completion of the perception and EM-1 tasks, participants performed an episodic memory task for items that were presented as lures during EM-1. The 80 Old pictures that served as lures during EM-1 were pseudo-randomized with 40 (20 emotional, 20 neutral) New lure pictures. Old and New pictures did not statistically differ in normative arousal and valence, other than between the emotional and neutral pictures – negative pictures were significantly more negative and arousing than neutral pictures. The average normative arousal and valence scores for Old and New emotional and neutral pictures for the second episodic memory task were as follows: 5.95/2.66 for emotional Old pictures; 5.91/2.46 for emotional New pictures; 3.41/5.02, for neutral Old pictures; and 3.52/5.00 for neutral New pictures.

### Experimental procedures

After the first experimental session (consisting of the perception and EM-1 tasks) participants were asked to return to the lab for further testing in one-week. The procedure for EM-1 is identical to that described in Shafer and Dolcos (2012). Approximately one-week later, 14 participants returned and completed EM-2 outside of the scanner. The test included a total of 120 pictures (80 Old, 40 New) distributed across 5 runs (24 pictures/run). To avoid mood induction, trials were pseudo-randomized such that no more than two consecutive trials of the same valence type occurred. As with EM-1, each picture was displayed for 2 s during which participants had to indicate with a button press whether the picture was Old, presented during EM-1 or New, not presented in EM-1. Immediately following the 2 s response window, a confidence rating screen was presented for 2 s asking participants to indicate the level of confidence (LOC) for their decision on a three-point Likert scale (1 = lowest, 3 = highest). Each trial was followed by a jittered fixation interval drawn from an exponential distribution with a median of 6 s (Range = 4–12 s). Similar to EM-1, participants were not informed of the EM-2 task. During both memory tasks participants were instructed to respond accurately and quickly, and that if they were unsure if a picture was Old or New to provide their best guess and indicate that their decision was uncertain by assigning that trial a low confidence rating.

### Imaging protocol

MRI data were collected on a 1.5-T Siemens Sonata scanner. After the sagittal localizer and the 3-D MPRAGE anatomical images (TR = 1600 ms; TE = 3.82 ms; FOV = 256 × 256 mm; number of slices = 112, voxel size = 1 mm^3^), EPI functional volumes allowing for full brain coverage were acquired axially (TR = 2000 ms; TE = 40 ms; FOV = 256 × 256 mm; number of slices/volume = 28, voxel size = 4 × 4 × 4 mm).

### Behavioral analyses

Responses in EM-1 and EM-2 tasks were classified into one of four categories [Hits – Old pictures correctly identified as Old; Misses – Old pictures incorrectly identified as New; CRs – New pictures correctly identified as New; False Alarms (FAs) – New pictures incorrectly identified as Old], as derived from signal detection theory (Macmillan and Creelman, 1991). These categories were used to calculate corrected recognition (% Hits – % FAs) scores. Since the main goal of the present investigation was to distinguish between the neural correlates of retrieval success and incidental encoding success during retrieval, all trials from the perception task were included in the data analysis for EM-1 (i.e., regardless of whether they were associated with correct or incorrect responses in the perception task). Previous analyses of this data set focused on brain imaging data acquired during the perception task and results focused on emotional distraction and encoding success for correct trials were published elsewhere (Shafer and Dolcos, 2012; Shafer et al., 2012). To maximize the difference in the MTL response during retrieval between remembered and forgotten items, only items that were given a LOC of 3 were included in the data analyses for both EM-1 and EM-2 tasks (Kleinsmith and Kaplan, 1963; Yonelinas, 2001; Daselaar et al., 2006). To assess the influence of emotion on memory performance, emotional and neutral corrected recognition scores were entered into a paired samples *t*-test. This was done separately for EM-1 and EM-2. To determine if differences in the delay period between EM-1 and EM-2 affected memory performance corrected recognition scores across the two tasks were examined using repeated measures analyses of variance (ANOVA) for the 10 participants who had both EM-1 and EM-2 data. Task (EM-1, EM-2), Valence (Emo, Neu), and Memory (Hit, Miss) were within-subject variables. *Post-hoc* comparisons were performed, where appropriate, and Bonferroni corrected.

### fMRI analyses

Statistical analyses were preceded by the following preprocessing steps (performed with SPM2 – Statistical Parametric Mapping): TR alignment, motion correction, normalization, and smoothing (8 mm kernel). For the data analysis, we used in-house custom MATLAB scripts involving both whole-brain voxel-wise and region-of-interest (ROI) analyses to compare brain activity associated with conditions of interest. For subject-level analyses, the fMRI signal was selectively averaged for each participant as a function of trial type (i.e., emotional hits, emotional misses, neutral hits, neutral misses, remembered emotional lures, forgotten emotional lures, remembered neutral lures, forgotten neutral lures) and time point (or TR; one pre- and 8 post-stimulus onset) using custom MATLAB scripts. Pair-wise *t* statistics for the contrasts of interest were calculated for each subject; no assumption was made about the shape of the hemodynamic response function (Dolcos and McCarthy, 2006; Dolcos et al., 2008; Morey et al., 2009). Individual analyses produced whole-brain activation *t* maps for each condition, contrast of interest, and TR/time point (TP). The outputs of the subject-level analyses were used as inputs for the second-level, random-effects within-group analyses.

### Region of interest (ROI) analyses

#### Identification of brain activity linked to retrieval success and the incidental encoding success of lure items

Of the fourteen participants that completed EM-2, only 10 participants met the criteria for inclusion when examining the number of trials per condition for LOC 3 responses. Criteria for inclusion required that each participant have at least five (Huettel and McCarthy, 2001) good trials (raw MR signal ≥ 300 MR units) per trial type, associated with high level of confidence (LOC 3) ratings. Thus, imaging data analyses assessing the neural correlates of retrieval success were performed on data from 17 participants, while imaging data analyses assessing the neural correlates of incidental encoding success were performed on data from 10 participants. To identify MTL activity linked to the memory-enhancing effect of emotion, analyses directly comparing brain activity between emotional and neutral retrieval/encoding success [i.e., (emotional vs. neutral Hits – Misses)/(emotional vs. neutral Remembered – Forgotten Lure Items), respectively] were performed on trials where behavioral differences were observed and where memory strength is the strongest (i.e., LOC 3 trials). Brain regions associated with emotional (Emotional Hits > Misses) and neutral (Neutral Hits > Misses) memory were separately identified for each time point. These contrasts for emotional and neutral retrieval/encoding success were then entered into a paired samples *t*-test which was then inclusively masked by the main effect of emotional retrieval/encoding success (Emotional Hits > Misses). This procedure allowed identification of MTL regions where activity for emotional retrieval/encoding success was greater than for neutral retrieval/encoding success, for each time point [i.e., ((Emotional Hits-Misses) vs. (Neutral Hits-Misses)) ∩ (Emotional Hits-Misses)]. The inclusive masking of the interaction (or conjunction analyses) identified greater retrieval success for emotional than for neutral pictures [i.e., (Emotional Hits > Misses) > (Neutral Hits > Misses)]. This was necessary to ensure that the interaction difference occurred in regions also showing significant retrieval success activity for the emotional pictures (Emotional Hits > Misses). This is a more conservative procedure in identifying differences between retrieval success activity for emotional and neutral material because it eliminates areas where such differences could be driven by the absence of retrieval success activity for emotional stimuli (e.g., if activity for Emotional Hits is not significantly greater than for Emotional Misses), coupled with effects going in opposite direction for the neutral pictures (Neutral Misses > Neutral Hits). To identify MTL regions where the response for neutral memory was greater than the response to emotional memory contrasts for neutral retrieval/encoding success were entered into a paired samples *t*-test which was then inclusively masked by the main effect of neutral retrieval/encoding success [i.e., ((Neutral Hits-Misses) vs. (Emotional Hits-Misses)) ∩ (Neutral Hits-Misses)].

Conjunction analyses involved masking procedures performed in MATLAB using the logical function AND. Thus, only voxels that met the threshold criteria in each of the contributing *t* maps survived the masking procedure. This procedure is consistent with the conjunction null hypothesis testing (Nichols et al., 2005). In addition, areas of activation were corrected for multiple comparisons in two ways. We applied two levels of false discovery rate (FDR) corrections, one corresponding to a *p*-value of 0.05 (Genovese et al., 2002) for each anatomical ROI, and the other corresponding to a *p*-value of 0.05 for each functional cluster within anatomically restricted ROIs (i.e., restricted to the anatomical boundaries of the MTL ROIs) – see Tables 1, 2. The present procedure involving FDR corrections and conjunction analyses, along with the reporting of both corrected and uncorrected statistical values offer a good balance between the cost of potential Type I and II errors (Lieberman and Cunningham, 2009). The greatest effect in MTL regions for emotional and neutral retrieval/encoding success occurred from time points 5–7 (6–10 s after stimulus onset). MTL activity for these time points was isolated using the Automated Anatomical Labeling atlas (AAL, Tzourio-Mazoyer et al., 2002) in SPM for the HC and PHC. These were used in conjunction with an in-house AMY mask (Dolcos et al., 2004; Moore et al., 2014) which corrected for large discrepancies in the AAL AMY mask.

**Table 1:** **MTL regions engaged in emotional and neutral retrieval success**.

**MTL regions**	**Hemisphere**	**Talairach coordinates**			***t*-values Cluster corrected**	**Cluster size**	**Time (s)**
		***x***	***y***	***z***			
**EMOTIONAL RS**							
AMY	L	−19	0	−14	6.17^¶^^*^	62	6
		−30	−8	−11	5.06^¶^^*^		6
		−23	−8	−11	4.58^¶^^*^	5	8
		−30	8	−17	**3.57**	24	8
		−19	4	−14	2.75^*^		8
	R	29	4	−13	**5.5^¶^**	46	6
		21	4	−17	**4.63^¶^**		6
		14	0	−10	**4.31^¶^**		6
		25	4	−17	**3.11**	15	8
HC	L	−23	−23	−9	5.38^¶^^*^	69	6
		−34	−11	−12	5.07^¶^^*^		6
		−19	−11	−12	3.92^¶^^*^		6
		−30	−11	−15	4.62^*^	59	8
		−19	−15	−12	**3.54**		8
		−16	−27	−6	**2.73**		8
		−30	−11	−15	2.69^*^	8	10
	R	29	−12	−7	**3.81^¶^**	69	6
		18	−4	−17	**3.35^¶^**		6
		21	−27	−5	**3.26^¶^**		6
		32	−20	−8	3.35^*^	10	8
PHC	L	−19	0	−18	6.18^¶^^*^	70	6
		−23	−22	−20	5.33^¶^^*^		6
		−16	−35	−6	**2.56^¶^**		6
		−16	4	−14	4.08^*^		8
		−31	−34	−10	**3.85**		8
		−27	−18	−20	3.3^*^		8
		−19	−27	−9	2.69^*^		8
	R	14	−3	−21	**3.66^¶^**	18	6
		14	4	−13	3.5^¶^^*^		6
		25	−31	−9	3.51^¶^^*^	22	6
**NEUTRAL RS**							
AMY	L	−23	−1	−7	2.51^*^	27	6
		−23	8	−21	2.41^*^		6
		−19	0	−14	**2.17**		6
		−16	−8	−11	3.74^*^	22	8
		−27	0	−14	2.49^*^		8
		−19	4	−14	1.89^*^		8
		−16	−4	−11	3.05^*^	35	10
		−27	−4	−15	2.8^*^		10
		−30	1	−21	2.69^*^		10
	R	18	−1	−6	2.84^*^	15	6
		21	0	−13	2.18^*^	10	8
HC	L	−31	−20	−5	**2.92**	5	6
		−30	−15	−12	4.07^*^	42	8
		−27	−11	−12	3.75^*^	22	10
		−30	−3	−18	**3.09**		10
	R	14	−35	1	3.06^*^	16	6
		21	−24	−5	**2.17**		6
		25	−39	1	**2.56**	11	8
		14	−31	−2	**2.27**		8
		18	−12	−11	2.43^*^	6	8
		21	−19	−12	1.97^*^		8
		32	−15	−11	1.96^*^	6	8
		32	−23	−8	1.86^*^		8
		21	−31	−2	**2.48**	7	10
		21	−39	1	**2.46**		10
		32	−12	−11	**2.26**	7	10
PHC	L	−16	4	−14	3.19^*^	10	6
		−34	−14	−23	**3.12**	5	8
		−16	−7	−15	3^*^	5	8
		−19	−23	−13	2.1^*^	5	8
		−23	−15	−16	2.07^*^		8
		−19	−23	−13	2.61	5	10
		−19	−15	−15	2.4		10
	R	21	−39	−3	**3.75**	6	8
		21	−39	−3	**3.27**	17	10
		18	−26	−16	3.26^*^		10

The table identifies MTL regions where emotional and neutral RS are significant. The displayed t-values correspond to the peak voxel for each time point after stimulus onset (where the HDR response was maximal), and represents a significant difference in MR signal between remembered and forgotten items. Emotional RS = (Emotional Hits-Misses) and Neutral RS = (Neutral Hits-Misses).

* *regions surviving exclusive masking by incidental encoding*.

¶ *region surviving FDR-anatomical ROI correction. MTL, medial temporal lobe; AMY, amygdala; HC, hippocampus; PHC, parahippocampus; L, left; R, right; RS, retrieval success; HDR, hemodynamic response; MR, magnetic resonance; Cluster Cor., False Discovery Rate-cluster correction. Cluster corrected values are indicated in bold*.

**Table 2:** **MTL regions specifically engaged in emotional vs. neutral retrieval success activity**.

**MTL regions**	**Hemisphere**	**Talairach coordinates**			***t*-values Cluster corrected**	**Cluster size**	**Time (s)**
		***x***	***y***	***z***			
**EMOTIONAL > NEUTRAL RS**							
AMY	L	−19	0	−14	3.84^*^	23	6
		−30	−8	−11	2.88^*^		6
	R	27	4	−13	**4.16**	5	6
		14	0	−17	**2.56**	6	6
HC	L	−23	−39	4	3.3^*^	27	6
		−30	−7	−15	2.62^*^		6
		−20	−27	−6	2.26^*^		6
	R	18	−11	−18	3.85^*^	28	6
PHC	L	−23	−22	−20	5.75^¶^^*^	6	6
		−19	−30	−10	3.61^*^	50	6
		−16	0	−12	6.32^*^		6
		−27	−22	−23	2.56^*^	7	8
	R	21	−11	−22	3.62^*^	36	6
		18	−34	−9	3.54^*^		6
		14	4	−13	2.61^*^		6
**NEUTRAL > EMOTIONAL RS**							
HC	R	25	−39	1	**2.87**	7	8
		21	−31	−2	**3.09**	7	10
PHC	R	18	−26	−19	2.92^*^	13	10
		17	−39	−3	2.38^*^		10

The table identifies MTL regions where emotional RS was greater than neutral RS and where neutral RS was greater than emotional RS. The displayed t-values correspond to the peak voxel for each time point after stimulus onset (where the HDR response was maximal), and represents a significant difference in MR signal between emotion RS and neutral RS. Emotional > Neutral RS = [((Emotional Hits-Misses)—(Neutral Hits-Misses)) ∩ (Emotional Hits-Misses)]. Neutral > Emotional RS = [((Neutral Hits-Misses)—(Emotional Hits-Misses)) ∩ (Neutral Hits-Misses)]. Extent threshold = 5 voxels.

¶ *regions surviving FDR-anatomical ROI correction*.

* *regions surviving exclusive masking by incidental encoding. MTL, medial temporal lobe; AMY, amygdala; HC, hippocampus; PHC, parahippocampus; L, left; R, right; RS, retrieval success; Cluster Cor., False Discovery Rate-cluster correction. Cluster corrected values are indicated in bold*.

#### Dissociating retrieval processes linked to the memory-enhancing effect of emotion

MTL activity observed across time points of greatest activity (time points 5–7), as identified in the individual analyses for incidental encoding success, was merged in MATLAB using the logical function OR. This was done separately for emotional and neutral items, to identify and collapse all significant areas of activation for each of the peak time points. For example, the clusters of activation identified in the MTL for emotional incidental encoding success (Emotional Lures Remembered-Forgotten) at time points 5, 6, and 7 were combined into one t-map representing MTL activity for emotional incidental encoding success and for the memory-enhancing effect of emotion incidental encoding success [((Emotional Lures Remembered-Forgotten) vs. (Neutral Lures Remembered-Forgotten)) ∩ (Emotional Lures Remembered-Forgotten)]. This same process was applied to *t* maps for neutral encoding success activity and neutral > emotional encoding success activity. Due to the low number of participants contributing to the analyses identifying incidental encoding success activity during retrieval, we implemented the incidental encoding success results as binary maps to identify areas showing retrieval success activity that have no contribution to incidental encoding success activity during retrieval. Specifically, we made the group *t*-maps identified by the steps mentioned above for emotional, neutral, emotional > neutral, and neutral > emotional incidental encoding success into binary masks. That is, voxels were assigned a value of 1 if *p*-values were less than or equal to 0.05, and all other voxels within the ROIs not meeting this criterion were given a value of zero. For each ROI, *t*-maps corresponding to emotional, neutral, emotional > neutral, and neutral > emotional retrieval success activity were then exclusively masked with this binary mask [e.g., (Emotional Hits > Misses) ~ (Emotional Lure Hits > Lure Misses), (Neutral Hits > Misses) ~ (Neutral Lure Hits > Lure Misses)], and regions surviving these masking procedures were identified (see Tables 1, 2). MTL activity for emotional, neutral, emotional > neutral, and neutral > emotional retrieval success *t*-maps after exclusively masking for activity associated with incidental encoding success was isolated using the AAL atlas in SPM for the HC and PHC. These were used in conjunction with an in-house AMY mask (Dolcos et al., 2004; Moore et al., 2014).

#### Exploratory analysis investigating possible temporal and spatial dissociations between emotional and neutral retrieval success

After exclusively masking by incidental encoding success activity during retrieval, functional ROIs from within region clusters for emotional greater than neutral retrieval success activity, and neutral greater than emotional retrieval success activity (e.g., right anterior PHC and right posterior PHC, respectively) were used to extract the fMRI signal for each participant, for each condition, and time point. These data were then entered into a repeated measures ANOVAs assessing Memory (Hits, Misses), Valence (Emotional, Neutral), and Time Point (5, 6, and 7). A significant three-way interaction in a region was further investigated at each time point for a Memory by Valence interaction. Follow-up *post-hoc* comparisons were performed, where appropriate, and Bonferroni corrected.

## Results

### Behavioral results

#### Increased memory for emotional pictures

**Figure 2:**
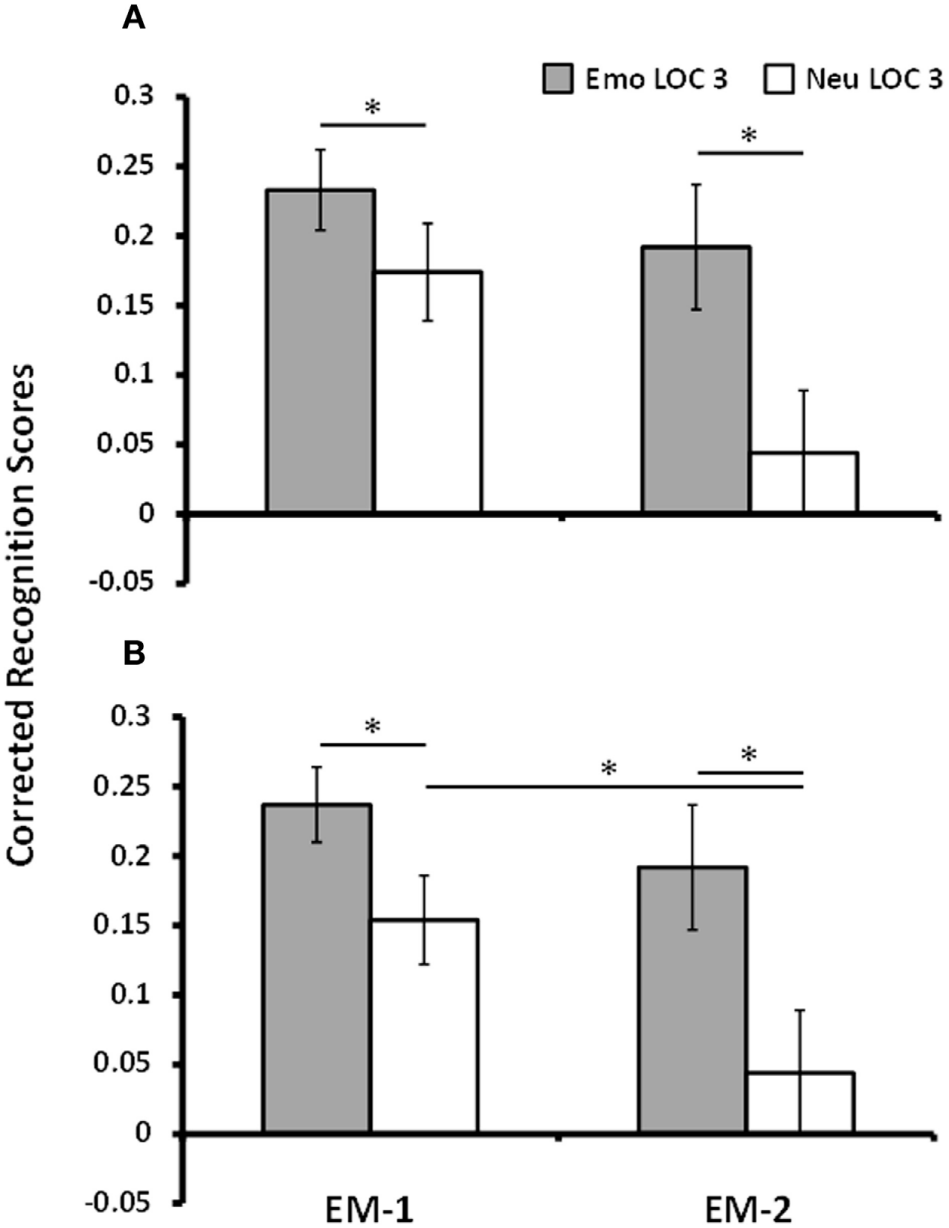
**Increased memory for emotional pictures**. Figure shows corrected recognition scores for Emo and Neu LOC 3 items for EM-1 (left) and EM-2 (right). Memory performance was greater for Emo items than for Neu items in EM-1 and EM-2 **(A)**, which was also identified when tested with the subset of participants that had both EM-1 and EM-2 data **(B)**. There was a significant decrease in memory performance for neutral items from EM-1 to EM-2. Emo, Emotional; Neu, Neutral; LOC, Level of Confidence; EM-1, Episodic Memory task 1; EM-2, Episodic Memory task 2. ^*^ indicates *p*-value < 0.05, one-tailed.

To maximize the difference in response to remembered and forgotten items in the brain imaging data, only trials that were given the highest level of confidence (LOC 3) were used for analyses (Kleinsmith and Kaplan, 1963; Yonelinas, 2001; Daselaar et al., 2006). Analyses of corrected recognition scores for LOC 3 trials for EM-1 showed that emotional pictures (*M* = 0.23, *SE* = 0.03) were better remembered than the neutral pictures (*M* = 0.17, *SE* = 0.04), [*t*_(16)_ = 1.85, *p* = 0.04, one-tailed]. Mean hit and FA rates for LOC 3 trials in the first episodic memory task for emotional/neutral pictures were as follows: 0.56 (*SD* = 0.13)/0.37 (*SD* = 0.18) and 0.33 (*SD* = 0.14)/0.19 (*SD* = 0.11), respectively. Similarly, analyses of corrected recognition scores for LOC 3 trials for EM-2 showed that memory for emotional lures (*M* = 0.19, *SE* = 0.05) was better than memory for neutral lures (*M* = 0.04, *SE* = 0.04), [*t*_(9)_ = 4.12, *p* = 0.001]. For the second episodic memory task mean hit and FA rates for emotional/neutral pictures were: 0.43 (*SD* = 0.12)/0.23 (*SD* = 0.11) and 0.24 (*SD* = 0.14)/0.19 (*SD* = 0.1), respectively. These analyses identified greater memory for emotional than neutral pictures in both EM tasks, with a numerically greater emotion effect following longer delay (Dolcos et al., 2005; Ritchey et al., 2008)—see Figure 2A. To investigate whether this interaction between emotional memory and EM task was significant a repeated measures ANOVA with the variables Task, Valence, and Memory was performed on the 10 participants who met the LOC 3 criterion for EM-2. The impact of emotion on memory was found to increase by 46% from EM-1 to EM-2. However, this increase across tasks was not statistically significant [*F*_(1, 9)_ = 1.44, *p* = 0.26]. Interestingly though, and consistent with research showing emotional memories persist better over time compared to neutral memory (Dolcos et al., 2005; Ritchey, 2008; Ritchey et al., 2008), this apparent change in the impact of emotion on memory across tasks was not due to an increase in emotional memory *per se* [*t*_(9)_ = 0.82, *p* = 0.43], but because of a decrease of the neutral memory in the second episodic memory task [*t*_(9)_ = 2.37, *p* = 0.04] – see Figure 2B.

### fMRI results

#### Dissociating retrieval processes linked to the memory-enhancing effect of emotion

To examine MTL activity associated with the memory-enhancing effect of emotion at retrieval, we first contrasted activity for emotional remembered and forgotten items, and neutral remembered and forgotten items. Increased activity throughout the MTL was identified in response to emotional and neutral memory (see Table 1). Next, to examine the memory-enhancing effect of emotion at retrieval, we contrasted activity for emotional and neutral memory [(Emo Hits > Misses) > (Neu Hits > Misses)]. Replicating previous findings of the involvement of MTL regions in the retrieval of emotional items (Dolcos et al., 2005), emotional compared to neutral retrieval success resulted in greater activity in bilateral AMY, HC, and PHC (see Table 2). When examining retrieval-related activity without accounting for activity related to incidental encoding success during retrieval, three clusters of activity were identified in the AMY. A left hemisphere cluster extended through the entire amygdala and two right hemisphere clusters, one located laterally and the other medially. Two clusters of activity were identified in the HC. A right hemisphere cluster localized more anteriorly and a left hemisphere cluster that extended the entire length of the HC and contained an anterior, middle, and posterior peak. The PHC contained two clusters of activity in response to emotional greater than neutral retrieval success, one left and one right. Both clusters extended throughout the PHC and contained three peaks: anterior, middle, and posterior.

**Figure 3:**
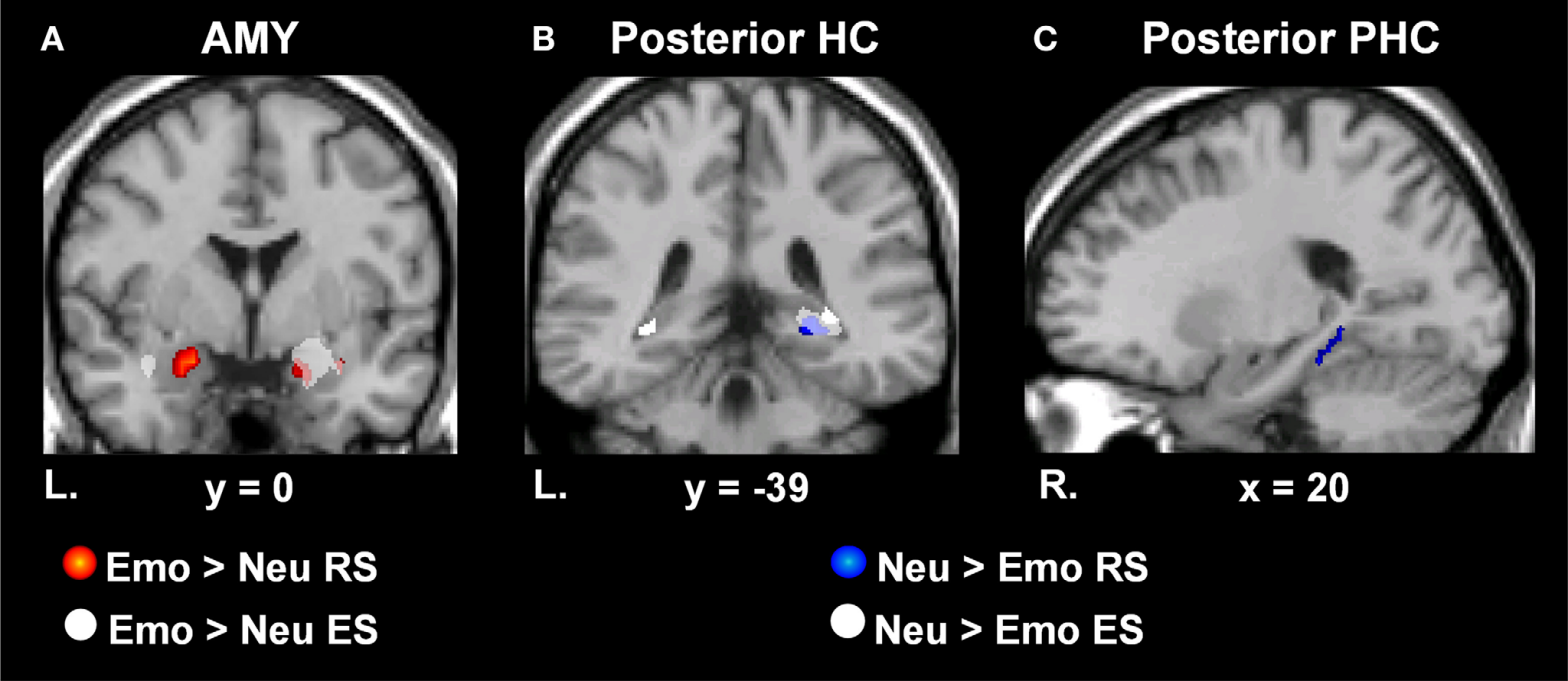
**Dissociating retrieval processes linked to the enhancement of emotional memory and increased engagement associated with neutral memory**. MTL regions sensitive to Emo vs. Neu RS (red) and incidental ES (white) of Emo vs. Neu lure items presented during EM-1. Bilateral AMY activity was identified for Emo > Neu RS, but activity in the right hemisphere was accounted for by incidental Emo > Neu ES activity **(A)**. MTL regions showing greater RS activity for Neu > Emo items (blue) and incidental ES activity for Neu > Emo lure items (white) presented during EM-1. Right HC tail activity was identified for Neu > Emo RS, but was accounted for by encoding-related activity for lures during retrieval **(B)**. RS activity identified in the right posterior PHC, was unaccounted for by encoding related activity **(C)**. Areas indicated in red illustrate the difference in activation in response to Emo > Neu RS, masked with the main effect of Emo RS. Areas indicated in white on panel **(A)** illustrate activation in response to Emo > Neu ES, masked with the main effect of Emo ES. Areas indicated in blue illustrate the difference in activation in response to Neu > Emo RS, masked with the main effect of Neu RS. The *t*-values correspond to the *t* map for Neu > Emo RS. Areas indicated in white on panels **(B,C)** illustrate the activation in response to Neu > Emo ES, masked with the main effect of Neu ES. These activation maps are superimposed on a high-resolution brain image displayed in coronal **(A,B)** and sagital **(C)** views. The Talairach x and y coordinates for the corresponding plane is indicated below each high-resolution brain image. AMY, Amygdala; HC, hippocampus; PHC, Parahippocampus; MTL, Medial Temporal Lobe; RS, Retrieval Success; ES, Encoding Success; Emo, Emotional; Neu, Neutral; L, Left; R, Right; EM-1, Episodic Memory task 1.

To investigate MTL activity dissociating retrieval success from incidental encoding successes during retrieval, linked to the memory-enhancing effect of emotion we exclusively masked retrieval activity by incidental encoding activity [((Emo Hits > Misses) > (Neu Hits > Misses)) ~ ((Emo Lures Remembered > Forgotten) > (Neu Lures Remembered > Forgotten))]. MTL retrieval success activity related to the memory-enhancing effect of emotion that survived exclusive masking by incidental encoding success activity related to the memory-enhancing effect of emotion was identified in the left AMY, bilateral HC, and PHC (see Table 2 and Figure 3A). For AMY, the two right hemisphere clusters identified for retrieval success also contributed to incidental encoding success. For the HC and PHC, partial activity within each of the clusters identified for retrieval success also contributed incidental encoding success during retrieval. However, all of the cluster peaks identified for retrieval success survived the exclusive masking procedure.

In addition, investigation of MTL areas with greater sensitivity to neutral than to emotional retrieval [(Neu Hits > Misses) > (Emo Hits > Misses)], identified the right posterior HC and PHC. To determine whether this retrieval-related activity contributed to the incidental encoding of neutral lure items, we exclusively masked retrieval-related by incidental encoding-related activity [((Neu Hits > Misses) > (Emo Hits > Misses)) ~ ((Neu Lures Remembered > Forgotten) > (Emo Lures Remembered > Forgotten))].

Activity surviving the exclusive masking procedure was located in the right posterior PHC (see Table 2 and Figures 3B,C). Right posterior HC activity was found to contribute to both retrieval and incidental encoding success of neutral lure items during retrieval.

#### Exploratory analysis investigating possible temporal and spatial dissociations between emotional and neutral retrieval success

**Figure 4:**
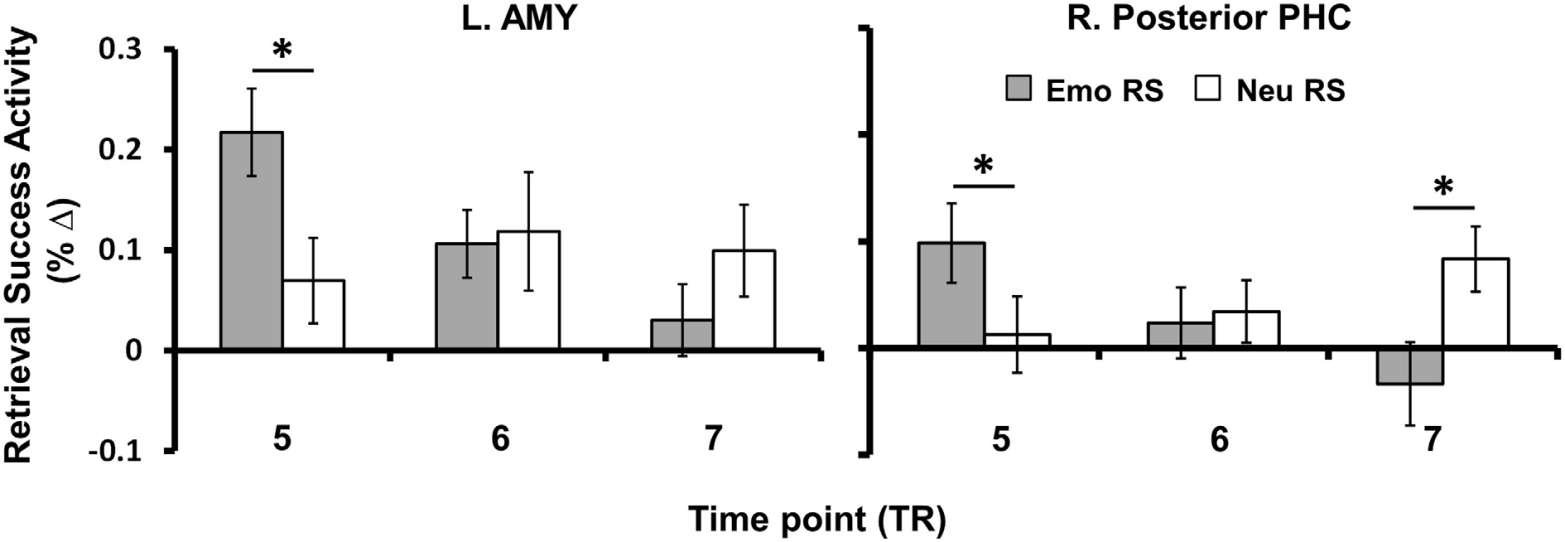
**Anterior-posterior and early-late dissociations identified for enhancement of Emotional vs. Neutral RS**. MTL regions linked to the enhancement of memory for emotional or neutral items (i.e., those surviving exclusive masking as shown in Table 2) showed spatial and temporal differences in the BOLD responses. Regarding the spatial dissociation, Emo > Neu RS was identified in anterior, middle, and posterior regions of the MTL (AMY, HC, and PHC), whereas Neu > Emo RS was identified only in posterior PHC. Regarding the early-late dissociation, MTL activity linked to memory enhancement for emotional items was found at an earlier time point after stimulus onset, whereas MTL activity linked to memory enhancement for neutral items occurred at a later time point. Bar graphs illustrates the fMRI signal as extracted from the AMY and posterior PHC clusters corresponding to the difference in activation between hits and misses for emotional and neutral items, for TP five (6–8 s post-stimulus onset), six (8–10 s post-stimulus onset), and seven (10–12 s post-stimulus onset). Asterisks indicate that the difference between Hits and Misses (i.e., RS) was significantly greater for emotional than for neutral conditions or vice-versa. Error bars represent standard errors of means. AMY, Amygdala; HC, Hippocampus; PHC, Parahippocampal Cortex; BOLD, Blood-Oxygen-Dependent-Response; RS, Retrieval Success; TP, Time Point; TR, Repetition Time; Emo, Emotional; Neu, Neutral; L, Left; R, Right; ^*^p < 0.05, two-tailed.

To examine differences in the temporal dynamics for retrieval processes linked to the enhancement of emotional memory vs. increased engagement for neutral memory (as observed in Table 2), we investigated changes in retrieval success activity over the peak time points of activation (time points 5–7). Sub-regions of the MTL sensitive to the enhancement of emotional memory or increased engagement for neutral memory that survived exclusive masking by encoding success activity during retrieval (see Table 2), showed earlier modulation for the enhancing effect of emotion (left AMY, bilateral HC and PHC) and later modulation for increased engagement associated with neutral memory (right posterior PHC) – see Figure 4. Regarding spatial dissociation, although greater emotional than neutral retrieval success activity was identified in AMY, HC, and PHG, regions showing greater neutral than emotional retrieval success activity were restricted to more posterior regions of the MTL (PHC) (see Table 2).

## Discussion

The present study used a novel experimental paradigm to dissociate between retrieval and incidental encoding success activity during emotional retrieval in the MTL. Two novel findings regarding the neural correlates of emotional retrieval were identified. First, greater emotional retrieval success activity was identified bilaterally in AMY, HC, and PHC. However, AMY activity was most impacted when accounting for encoding success activity, as only retrieval success activity in the left but not right AMY was dissociated from encoding success activity during retrieval, whereas the portions of HC and PHC showing greater emotional retrieval success activity were largely uninvolved in encoding success. Second, an earlier and more anteriorly spread response (in left AMY and bilateral HC and PHC) was linked to greater emotional retrieval success, whereas a later and more posteriorly localized response (in right posterior PHC) was linked to increased engagement associated with neutral retrieval success. These findings are discussed in turn below.

### Dissociating retrieval processes linked to the memory-enhancing effect of emotion

Memory performance for emotional relative to neutral items for EM-1 and EM-2 replicates findings from a large body of extant research examining item-based emotional memory (for review, see Dolcos et al., 2012; Chiu et al., 2013). Although the impact of emotion on memory for EM-1 is numerically weaker than that found for EM-2, specific aspects of the present study's design can account for this difference. First, EM-1 tested memory for items that were presented as task-irrelevant distracters during initial encoding. Thus, divided attentional resources between processing these task-irrelevant items and those required for performing the main perceptual task most likely resulted in a decrease in overall memory performance (Uncapher and Rugg, 2005). Furthermore, the emotional distracter items that served as memoranda in EM-1 were most difficult to ignore during the perception task. As a result, participants may have engaged mechanisms to minimize their influence on the perception task more than those required for performing the perception task in the presence of neutral distraction. Hence, increased engagement of these mechanisms might have also contributed to the decreased impact of emotion on memory, compared to EM-2. Second, although expected to be effective (Kleinsmith and Kaplan, 1963; Dolcos et al., 2004), our short retention interval (~40 min) between encoding and retrieval of a single item also made a difference compared to the longer retention interval for EM-2 (Dolcos et al., 2005; Ritchey et al., 2008). Considering these aspects of the present design, the behavioral effect observed in EM-1 highlights the robustness of emotion's impact on memory, as it was present in conditions where the memoranda were task-irrelevant during initial encoding and when the retention interval was short.

Our result showing the persistence of emotional memory over time is also consistent with a number of earlier behavioral and neuroimaging studies (Kleinsmith and Kaplan, 1963; Sharot and Phelps, 2004; Dolcos et al., 2005; Anderson et al., 2006; Ritchey et al., 2008; Sharot and Yonelinas, 2008). Enhanced memory performance for emotional relative to neutral items was associated with increased engagement of all three main regions of the MTL (AMY, HC, and PHC) during retrieval, which replicates previous neuroimaging studies of emotional retrieval (Sharot et al., 2004; Dolcos et al., 2005; Kensinger and Schacter, 2005; Sergerie et al., 2006; Smith et al., 2006).

The first novel finding of the present study involved the identification of MTL sub-regions that survived exclusive masking by incidental encoding success processes co-occurring during retrieval. When considering this finding in the context of the various aspects of processing that occur during retrieval (including retrieval operations *per se*, re-encoding/consolidation of retrieved memories, and incidental encoding of new information presented as lures), the current investigation offers insight into MTL sub-regions that may distinguish between retrieval operations and other memory operations occurring during retrieval (specifically, the encoding of new information presented during retrieval). As mentioned earlier, encoding operations can refer to general perceptual processing or to mnemonic processing that leads to the formation of new memories. As implemented here, one way of distinguishing between incidental encoding activity during retrieval from activity specifically linked to retrieval, while controlling for repeated exposure effects is to (1) define retrieval success as the difference in activity between Hits and Misses, and (2) compare activity for remembered vs. forgotten New items that were presented as lures during retrieval. This approach allows for the comparison of brain activity related to memory operations involved in successful retrieval of Old items, to brain activity related to memory operations involved in successful encoding of New items during retrieval. Using this approach, we found greater emotional retrieval success activity in bilateral AMY, HC, and PHC. However, AMY activity was most impacted when accounting for encoding success activity, as only retrieval success activity in the left but not right AMY was dissociated from encoding success activity during retrieval, whereas the portions of HC and PHC showing greater emotional retrieval success activity were largely uninvolved in encoding success.

Retrieval processes have long been thought to involve the reactivation of encoding activity (Nyberg et al., 2000; Johnson and Rugg, 2007) and neuroimaging research has provided much support for this idea, with numerous studies showing that successful memory performance is associated with encoding-retrieval overlap in activations (Nyberg et al., 2000; Wheeler et al., 2000; Johnson and Rugg, 2007; Johnson et al., 2009; Ritchey et al., 2013). Moreover, emotional arousal has been shown to strengthen the relationship between memory performance and encoding-retrieval overlaps (Ritchey et al., 2013). Thus, it is reasonable to expect that successful encoding and retrieval of emotional memories involves similar mechanisms. However, the dissociation in MTL mechanisms during retrieval linked to retrieval success from those linked to encoding success demonstrate that it is possible to clarify the MTL's involvement in different memory operations that occur during recognition memory tasks. Open questions remain, however, regarding the mechanisms involved in the re-encoding/consolidation of emotional memories during retrieval (Nader and Einarsson, 2010).

### Exploratory analysis investigating possible temporal and spatial dissociations between emotional and neutral retrieval success

The second novel finding from the current study identified MTL regions where differences in the temporal dynamics of the BOLD response were found, with an earlier response to emotional enhancement of memory (left AMY, bilateral HC, and PHC) and a later response to increased engagement associated with neutral memory (right posterior PHC). This finding provides support for earlier research showing that the neural mechanisms subserving encoding of emotional memories are more quickly engaged than for neutral information (Dolcos and Cabeza, 2002; Larson et al., 2006; Mendez-Bertolo et al., 2013). Importantly, the current data extend these findings by showing that similar differences in timing are also present at retrieval. It is well known that emotion enhances the magnitude of early and late neural markers of stimulus processing, as shown by ERP studies (Olofsson et al., 2008), although shifts in the latency of ERP components indexing sensory and cognitive processes influenced by emotion are not commonly found (e.g., though this may be the result of event-related averaging vs. single-trial variability for ERP data). Also, negative emotions can produce faster responses of the oculomotor system resulting in quicker localization of threat (Bannerman et al., 2009, 2012). As also shown in the present results, fMRI studies of emotion processing can show differences in the temporal dynamics of the BOLD response between experimental conditions. These differences account for variance that would otherwise remain unexplained. However, these results should be treated with caution given the relatively poor temporal resolution of fMRI. More light will be shed on this topic as neuroimaging techniques advance to allow for the direct comparison and/or integration of more superior temporal techniques with more superior spatial techniques (e.g., simultaneous recordings of EEG and fMRI).

We also identified activation within the MTL showing greater neutral compared to emotional retrieval success. This activation was restricted to posterior HC and PHC regions. Only one cluster, right posterior PHC, survived exclusive masking by encoding success activity. Posterior MTL involvement in neutral greater than emotional retrieval success is consistent with previous research showing an anterior (emotional)—posterior (neutral) dissociation in the MTL during emotional memory encoding (Dolcos et al., 2004; Dougal et al., 2007) and retrieval (Sharot et al., 2004; Kensinger and Schacter, 2005). The reasons for this anterior-posterior dissociation remain unknown, but it has been suggested that this dissociation may be linked to the location relative to the AMY, with the anterior memory-related MTL regions being closer to the AMY and thus more involved in emotional memory due to rich interconnections between the AMY and anterior HC/PHC. The involvement of anterior MTL during emotional memory retrieval is consistent with the modulation hypothesis (McGaugh, 2004) of emotional memory encoding and consolidation, and with evidence from previous studies of emotional retrieval. The modulation hypothesis suggests that AMY exerts neuromodulatory influences on other brain regions (e.g., the MTL memory system) involved in memory formation. Our result showing more anterior MTL engagement during emotional memory retrieval is consistent with earlier reports on emotional retrieval (Dolcos et al., 2005; Kensinger and Schacter, 2005; Sergerie et al., 2006; Smith et al., 2006), where the functional interactions supported by the modulation hypothesis for encoding and consolidation are extended to retrieval (LaBar and Cabeza, 2006). While the neurobiological mechanisms underlying AMY-MTL memory system functional interactions during retrieval remain largely unclear, they have begun to be elucidated by psychopharmacological and animal studies. For instance, research showing the β-adernergic blockade of the memory-enhancing effect of emotion during retrieval (Kroes et al., 2010) and increased synchronization between AMY and HC during fear retrieval (Seidenbecher et al., 2003) suggest that the neuromodulatory role of the AMY in emotional memory is similar for encoding, consolidation, and retrieval. This is consistent with the current findings showing overlap between retrieval success and encoding success activity during retrieval. However, and as shown here, research also suggests that certain aspects of the processes occurring during retrieval can be dissociated (Stark and Okado, 2003), which is consistent with evidence from animal research (Nader and Einarsson, 2010). On the other hand, posterior HC/PHC regions due to their more remote location from the AMY may not be as susceptible to its modulatory influences, and hence their involvement in neutral memory is not “overshadowed” by emotion's influence. Instead, these areas are closer to regions associated with visual processing, and thus are perhaps more involved in item processing that engages the ventral visual stream (Ungerleider, 1995; Dolcos et al., 2004). Although the current findings show some posterior HC and PHC involvement for the memory-enhancing effect of emotion, the majority of MTL regions identified for this analysis were more anteriorly located, whereas increased MTL engagement for neutral relative to emotional memory was restricted to posterior regions. Future research is needed to clarify the causes of this dissociation.

### Caveats

A limitation of the present study is the number of participants contributing to the incidental encoding success analyses. Equal numbers of participants contributing to both retrieval success and encoding success analyses would have been ideal in providing increased specificity when dissociating MTL activity linked to different memory processes during retrieval. Therefore, although the present investigation allowed for identification of areas within the MTL specifically contributing to retrieval success by dissociating them from those also contributing to incidental encoding success during retrieval, the latter findings should be treated with caution. A high degree of specificity within the MTL in the current study is also limited due to data acquisition and processing parameters. Anatomical specificity is impeded when examining small areas of neural tissue using 4 mm isotropic voxels for data acquisition along with normalization and smoothing kernel of 8 mm for data processing, although these parameters are not unusual for fMRI studies. Future studies using higher spatial resolution and minimal preprocessing will allow for more precise specificity when examining this issue in the MTL. Another limitation of the current study is the difference in delay between encoding and retrieval for EM-1 and EM-2. In EM-1 there were approximately 40 min separating the encoding and retrieval of an item, whereas in EM-2 there was approximately 1 week separating encoding and retrieval. This difference in delay could have resulted in a shift in retrieval processes that were used during the memory tasks (e.g., from recollection-based for EM-1 to familiarity-based for EM-2). Thus, it is possible that the current findings could be influenced by differences in the retrieval processes implemented across the two memory retrieval tasks. On the other hand, previous research suggests that recollection-based retrieval processes are associated with the highest LOC (Daselaar et al., 2006). Since we selected only trials with the highest LOC ratings for analysis in both memory tasks there is increased likelihood that memory for these trials is recollection-based in both memory tasks. Moreover, extant research on emotional memory shows that the enhancing effect of emotion results from recollection rather than familiarity-based memory processes (Dolcos et al., 2005; Dew et al., 2014). Examination of the relationship between the engagement of certain retrieval mechanisms for items tested during retrieval (e.g., recollection and familiarity) and those engaged for items incidentally encoded during retrieval, linked to the retention interval, is an open question for future research.

## Conclusions

In summary, the present study yielded two novel findings pertaining to the neural mechanisms of emotional memory retrieval. First, the study distinguished between MTL retrieval and incidental encoding success activity linked to the memory-enhancing effect of emotion during retrieval. Greater emotional retrieval success was identified bilaterally in AMY, HC, and PHC. However, AMY activity was most impacted when accounting for encoding success activity, as only retrieval success activity in the left but not right AMY was dissociated from encoding success activity during retrieval, whereas the portions of HC and PHC showing greater emotional retrieval success activity were largely uninvolved in encoding success. This finding demonstrates that MTL activity during retrieval can be dissociated and linked to different memory operations that occur during recognition memory. Second, MTL sub-regions were identified as showing different temporal and spatial dissociations in the BOLD response for the memory enhancement of emotional items vs. increased engagement associated with the memory for neutral items. An earlier and more anteriorly spread response (in left AMY and bilateral HC and PHC) was linked to greater emotional retrieval success, whereas a later and more posteriorly localized response (in right posterior PHC) was linked to greater engagement for neutral retrieval success. Taken together, these results shed light on the neural mechanisms of emotional memory retrieval in healthy behavior and are important for understanding maladaptive alterations in the processes subserving emotional memory found in populations with affective disorders (Whalley et al., 2009; Hayes et al., 2011; Dolcos, 2013).

### Conflict of interest statement

The authors declare that the research was conducted in the absence of any commercial or financial relationships that could be construed as a potential conflict of interest.
